# An unfrecuenty case of Auditory Charles-Bonnet syndrome

**DOI:** 10.1192/j.eurpsy.2025.2048

**Published:** 2025-08-26

**Authors:** E. S. Gisbert, M. J. A. Abildua

**Affiliations:** 1 PSYCHIATRY/NEUROLOGY, HOSPITAL UNIVERSITARIO INFANTA SOFIA, SAN SEBASTIAN DE LOS REYES, Spain

## Abstract

**Introduction:**

In 1760, Charles Bonnet, a Genoese naturalist and philosopher, described the case of his grandfather, who experienced vivid, elaborate, and recurrent visual hallucinations and who also suffered from visual impairment. Bonnet himself later developed visual impairment and experienced similar symptoms. Since then, there have been multiple reports and cases in the European literature regarding this syndrome.

**Objectives:**

Auditory Charles-Bonnet syndrome describes a rare condition presenting with sensorineural hearing loss, which can result in auditory-musical hallucinations in the absence of an acoustic stimulus. It has been reported in patients with diseases such as psychiatric disorders and organic brain diseases. However, the most common are idiopathic musical hallucinations that occur along with deafness in elderly people. Musical hallucinations that accompany hearing loss may reflect impaired brain function.

**Methods:**

We present the case of a 84-year-old woman with a long-standing history of depression, who also presents mild bilateral pantonal sensorineural hearing loss with associated subjective tinnitus, without other associated somatic and/or psychiatric symptoms. In addition, a CT study of the head was performed which revealed severe fronto-temporal cortical atrophy.

**Results:**

The treatment remains the subject of extensive research. Some authors have reported that hearing aids, antiepileptic drugs, benzodiazepines and antipsychotics can alleviate musical hallucination, which in the case of our patient was eradicated, so the contribution of this case could enrich the current bibliography.

**Conclusions:**

This is unfrecuently presentation of Charles Bonnet symdrom.

**Disclosure of Interest:**

None Declared

